# Effects of Plant-Derived Dietary Supplements on Skin Health: A Review

**DOI:** 10.7759/cureus.40892

**Published:** 2023-06-24

**Authors:** Alyssa Abdelnour, Taylor Adlam, Geoffrey A Potts

**Affiliations:** 1 Department of Dermatology, Michigan State University College of Human Medicine, Grand Rapids, USA; 2 Department of Dermatology, Wayne State University, Detroit, USA

**Keywords:** dermatology, health, skin, supplements, oral, derived, plant

## Abstract

Dietary supplements have become increasingly popular to improve facial appearance and optimize skin health. With countless supplements available online and in stores, there are unlimited options for patients to choose from. Federal law does not require the Food and Drug Administration to assess each product’s efficacy before its appearance on the market. Therefore, evidence-based medicine is vital for dermatologists to provide adequate recommendations regarding the safety and efficacy of various dietary supplements. The goal of this review is to evaluate plant-derived, antioxidant oral supplements and their effects on wrinkle appearance, skin hydration, skin elasticity, and photoprotection.

## Introduction and background

Oral supplements are sought out to maintain skin health and diminish signs of aging. There has been an increase in aesthetic-based marketing with various over-the-counter supplements for skin health. Many oral supplements are often chemically synthesized or isolated from botanical extracts. The Food and Drug Administration (FDA) regulates dietary supplements differently than “conventional” foods and drug products, meaning these products do not need approval from the FDA before they are marketed and are not required to prove safety or efficacy [1]. Therefore, evidence-based medicine is needed for dermatologists to adequately counsel patients regarding safe and effective oral supplements. Currently, there are minimal reviews examining plant-derived, oral supplements and their effects on skin health. This review will explore the efficacy and effects of plant-derived, antioxidant supplements on skin parameters, including photoprotection, pigmentation, texture, wrinkles, hydration, elasticity, and microcirculation.

This article was previously presented at the American Academy of Dermatology Annual Meeting on March 17, 2023.

## Review

Methodology

**Figure 1:**
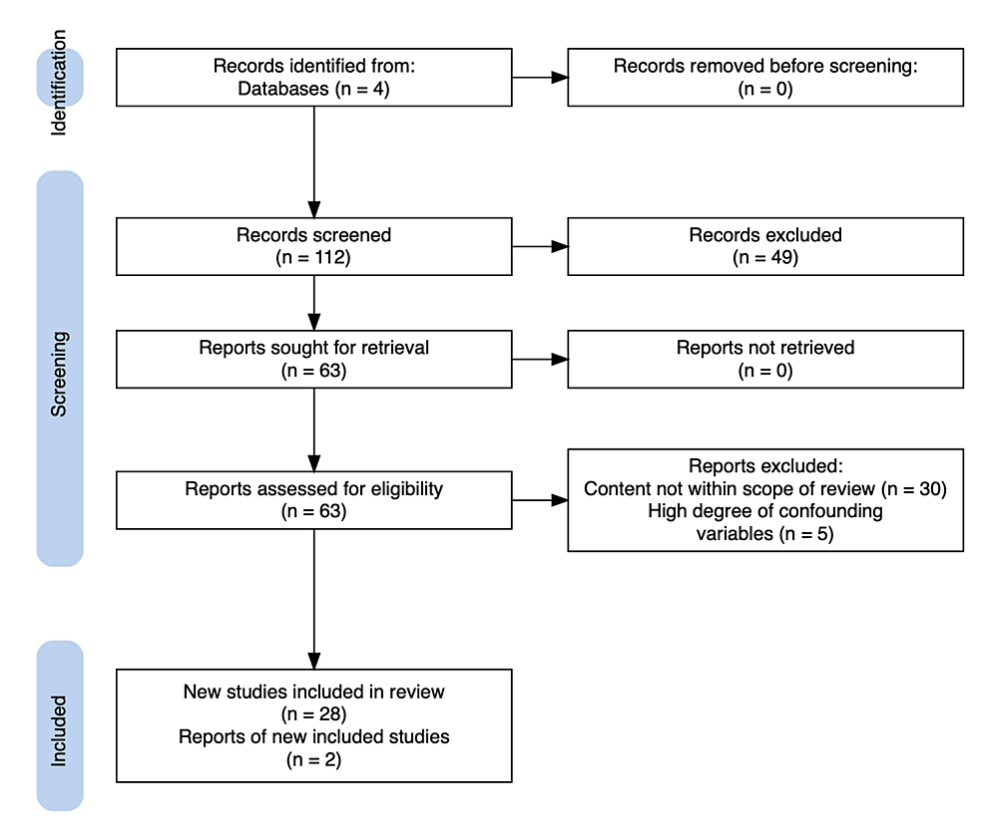
Identification and selection of included studies.

A comprehensive electronic search conducted from October 2021 to June 2023 with search terms “plant-derived oral supplements effects on skin” in PubMed/MEDLINE, Cochrane Central, Embase, and Google Scholar yielded 112 articles. Duplicate, non-English-language articles and articles not focused on our review were excluded. In total, 84 articles were excluded because they did not meet the search criteria. Two new articles were added. Therefore, 30 articles were finally included in this review (Figure 1).

Polyphenols

Signs of aging occur after an accumulation of molecular damage within cells and tissue of the skin. Oxidative stress can lead to skin deterioration under normal conditions, and its rate of damage increases with aging due to attenuated antioxidant and repair mechanisms [2].

Substances are described as an antioxidant if their presence delays or prevents oxidative damage within cells. Plant-derived antioxidants are organized into vitamins, polyphenols, carotenoids, small antioxidant molecules, enzymes, and trace elements.

Polyphenols are naturally occurring compounds found in fruits, vegetables, and legumes and are known to exhibit antioxidant properties as they play an integral role in plants’ defense against ultraviolet (UV) radiation and pathogen damage [3].

Polyphenols are divided into flavonoids and non-flavonoids. Subclasses of flavonoids are isoflavones, flavanols, flavanones, flavan-3-ols, flavones, and anthocyanidins. Flavan-3-ols are present as catechins (monomers) or proanthocyanidins (polymers).

Non-flavonoids can be further categorized into several groups, such as phenolic acids (gallic acid), hydroxycinnamic acids, and stilbenes. Caffeic and ferulic acids are often found as ester derivatives of hydroxycinnamic acids.

Flavonoids

French Maritime Pine Bark Extract

French maritime pine bark extract (PBE) is a standardized mixture derived from *Pinus pinaster maritima* and *aiton*. This extract contains monomers of catechins and epicatechins, flavonoid procyanidins and proanthocyanidins, and phenolic acids, such as caffeic, ferulic, and p-hydroxybenzoic acids [4]. Two popular supplements primarily made of PBE are Flavangenol and Pycnogenol.

PBE is photoprotective by reducing UV radiation damage through antioxidant and anti-inflammatory effects [4]. Age spots induced by photodamage are diagnosed as “solar lentigines,” which are associated with mottled pigmentation and keratosis. Significant improvement in solar lentigines was found after high-dose supplementation of PBE (100 mg Flavangenol tablet daily) for 12 weeks (n = 24) compared to the low-dose (40 mg daily) 12-week supplementation group (n = 38) and placebo (n = 39). Both treatment groups showed significantly lower scores for solar lentigines and skin roughness compared to the untreated group, indicating that PBE may reduce facial signs of photoaging [4].

Moreover, Saliou et al. [5] found oral Pycnogenol tablet supplementation of 1.1 mg/kg daily for four weeks and 1.66 mg/kg body weight per day for the following four weeks significantly increased minimal erythema dose (MED) (n = 21), suggesting photoprotective effects of the extract against solar UV-induced erythema.

Not only is there evidence of PBE supplementation displaying photoprotection but this supplement may also improve skin hydration and elasticity. Marini et al. found supplementation with 25 mg Pycnogenol capsules three times daily for 12 weeks significantly improved hydration and skin elasticity in 20 healthy postmenopausal women. In addition, supplementation with PBE increased mRNA expression of hyaluronic acid synthase-1 (HAS-1) suggesting extracellular matrix components, hyaluronic acid and collagen, could be playing a role in skin improvement [6].

Furthermore, in a study conducted by Zhao et al. [7], 12 weeks of 50 mg Pycnogenol capsule was taken twice a day (n = 39). It prevented a decrease in skin hydration, transepidermal water loss (TEWL), and skin darkening. Moreover, this supplement improved skin elasticity compared to placebo capsules containing cellulose (n = 37).

Capsule summary: Evidence supports PBE has photoprotective effects and can improve changes in skin pigmentation, texture, hydration, and elasticity.

Cocoa

Cocoa extract derived from the seeds of *Theobroma cacao* is naturally rich in flavanols, which may contribute to its beneficial effects.

Oral tablet supplementation of flavanol-rich cocoa (n = 10) 4-6 g/day (based on body weight) for one week showed a significant increase in MED compared to subjects (n = 6) taking 1 g/day, suggesting photoprotection as a result of supplementation [8]. Additionally, high-concentration flavanol cocoa beverage supplementation of 329 mg/day (n = 12) has been shown to also have photoprotective effects by increasing the MED compared to the low-concentration 27 mg/day supplementation (n = 12) after six and 12 weeks [9].

In the same study, the high-flavanol group improved cutaneous and subcutaneous blood flow, increased skin density and thickness, decreased skin roughness and scaling, increased skin hydration, and decreased TEWL compared to placebo. Smoothness and wrinkles did not change for either intervention group [9].

Neukam et al. also demonstrated that 329 mg of cocoa flavanols within a beverage (n = 5) increased cutaneous blood flow and oxygen saturation only two hours after one supplementation compared to 27 mg of flavanols (n = 5) [10].

Moreover, 4 g of cocoa powder (yielding 320 mg of total flavanols) was dissolved in 150-200 mL of hot water and consumed daily for 24 weeks. After 24 weeks of supplementation (n = 31), wrinkle depth, skin elasticity, and MED were improved compared to placebo (n = 31). However, there were no significant beneficial effects of supplementation on skin hydration (TEWL) or integrity of the skin barrier [11].

Capsule summary: Evidence supports supplementation with high-flavanol cocoa extract improves photoprotection and microcirculation.

Turmeric

*Curcuma longa*, also known as the herb turmeric, has been used in dermatological conditions for thousands of years due to its anti-inflammatory and antioxidant activity. Curcumin is an active poly-phenolic compound found within turmeric [12], its flavonoid and anthocyanin components have been found to largely contribute to the herb’s antioxidant activity [13].

While turmeric has been known for its antioxidant function, supplementation of four 500 mg tablets twice a day (total of eight tablets = 4,000 mg) did not have a significant effect on TEWL (n = 10) compared to placebo (n = 9) in a prospective, double-blind, randomized pilot study [14].

Capsule summary: There are minimal studies supporting significant changes in photoprotection, skin pigmentation, texture, wrinkle appearance, hydration elasticity, or microcirculation with turmeric supplementation.

Polypodium leukotomos Extract

*Polypodium leucotomos* is an extract derived from the fern family Polypodiaceae found in Central and South America. This extract is rich in phenolic compounds, including ferulic and caffeic acid which contribute to its antioxidant properties [15].

Three months of supplementation with a fixed *P. leucotomos*/pomegranate combination called PPmix (n = 20) and 480 mg/day of *P. leucotomos* alone (n = 20) were found to both increase skin hydration and elasticity [16]. While the erythema index was significantly decreased by both treatment groups, the fixed combination was more effective. Moreover, the melanin index and skin sebum content were decreased by the combination treatment, while the *P. leucotomos* extract (PLE) group alone did not have any significant changes.

Kohli et al. demonstrated a reduction in erythema and pigmentation within two hours of administration of 240 mg PLE (n = 22) [17]. Mohammad et al. further established how subjects (n = 22) supplementing 480 mg of PLE daily significantly decreased persistent pigment darkening and delayed tanning when exposed to visible light [18]. Through a reduction of molecular skin damage induced by free radicals, supplementation of PLE demonstrates photoprotective effects.

Capsule summary: There is evidence to support that supplementation with PLE improves photoprotection, skin hydration, and elasticity.

Non-flavanoids

Ferulic Acid

Ferulic acid (FA) is a component of plant cell walls and displays antioxidant functions. While topical application of FA has been shown to suppress the skin’s inflammatory reaction to UV radiation [19], there is limited data regarding its effects from oral supplementation.

In a double-blind, placebo-controlled, parallel pilot study, dietary supplementation of three 67 mg FA capsules per day (n = 8) for two weeks significantly decreased TEWL, improved subcutaneous hydration, enhanced flow-mediated dilatation, and enhanced skin barrier function compared to the placebo group (n = 8) [20].

Capsule summary: There is minimal evidence displaying improved hydration and microcirculation with supplementation of oral FA.

Carotenoids

While carotenoids are pigments used in photosynthesis and the coloration of plants [21], human skin also contains these molecules. The chemical structure of carotenoids is composed of a tetraterpene backbone, some with oxygenated or deoxygenated terminating rings. Six major carotenoids have been studied, including α-carotene, β-carotene, lycopene, lutein, β-cryptoxanthin, and zeaxanthin.

Astaxanthin

Astaxanthin is a red carotenoid found in shrimp, crab, salmon, and microalgae which exerts a strong antioxidant activity by scavenging free radicals [22].

In a randomized, double-blind, placebo-controlled, parallel-group comparison trial, astaxanthin’s effects on UV-induced skin deterioration were investigated in healthy Japanese subjects. One capsule of 4 mg astaxanthin (n = 12) or placebo (n = 11) was given for nine weeks. After supplementation, the treatment group showed a significant increase in MED and reduced loss of moisture in an irradiated area compared to the placebo group. Subjective skin conditions “for improvement of rough skin” and “texture” were significantly improved in the treatment group [22].

Tominaga et al. examined in two clinical studies how oral supplementation with AstaREAL Oil capsules, containing 5% astaxanthin (3 mg) *H. pluvialis* extract and canola oil, compared with the placebo which contained only canola oil. In study 1, two capsules were given twice daily to females for eight weeks (n = 30) along with 1 mL of topical application twice daily on the face. In study 2, astaxanthin (n = 18) and placebo capsules (n = 18) were given twice daily to men for six weeks. In study 1, significant improvements were seen in skin wrinkle, age spot size, elasticity, and moisture content. In study 2, significant improvements were observed in the wrinkle appearance and elasticity of the crow’s feet and TEWL [23].

Capsule summary: Evidence supports improvements in photoprotection, pigmentation, wrinkle appearance, hydration, and elasticity with astaxanthin supplementation.

Lutein, Zeaxanthin, and Lycopene

Lutein and zeaxanthin are carotenoid isomers that protect the skin from high-energy sources. Juturu et al. examined how 10 mg lutein and 2 mg zeaxanthin capsules (n = 25) once daily for 12 weeks affect skin health compared to a placebo group (n = 25). Capsules from the treatment group were derived from the extract of dried flowers of marigold tagetes spp., while placebo group capsules were made of safflower oil. After supplementation, overall skin tone and luminance were significantly increased in the treatment group compared to the placebo. Furthermore, MED was increased in the supplementation group compared to the placebo [24].

Grether-Beck et al. investigated the supplementation of lycopene-rich capsules and lutein capsules for 12 weeks. The lycopene capsules contained 5 mg lycopene, as well as other tomato phytonutrients, such as phytoene and phytofluene, tocopherols, and phytosterols. The lutein capsules contained 10 mg of free lutein stabilized by 10% carbonic acid. The placebo capsules contained soybean oil. The groups studied were the lycopene active group (TGA) (n = 15), lycopene placebo group (TGB) (n = 16), lutein active group (TGC) (n = 14), and lutein placebo group (TGD) (n = 14). Supplementation of lycopene-rich and lutein-containing capsules was associated with a significant decrease in UV radiation-induced mRNA expression of *HO-1*, *MMP-1*, and *ICAM-1*, which are radiation-inducible genes, thus providing evidence that lycopene and lutein may protect against radiation-induced skin damage [25]. Tarshish et al. found oral supplementation with 15 mg soft gels of lycopene (n = 50) resulted in significant improvement in TEWL, skin tonality, wrinkle appearance, pore size, and skin firmness after 12 weeks [26].

Schwartz et al. examined how supplementation with zeaxanthin (ZO-1), zeaxanthin with gel serum (ZO-2 + ZT), and placebo for 12 weeks affected skin health. ZO-1 supplementation contained zeaxanthin, sea buckthorn fruit oil, wheat ceramides, alpha-lipoic acid, green tea, red clover leaf, gotu kola seed, maritime pine bark, and vitamins C, E, and D3. The ZO-2 + ZT group contained zeaxanthin, sea buckthorn fruit oil, wheat ceramides, alpha-lipoic acid, green tea, red clover leaf, gotu kola seed, maritime pine bark, and vitamins C, E, and D3. Topical gel serum contained zeaxanthin, laminaria algae, porphyra algae, perfluorodecalin, sodium hyaluronate, chlorella, L-ergothioneine, FA, and tocopherol. The placebo group contained safflower oil. In both ZO-1 (n = 13) and ZO-2 + ZT (n = 10) groups, significant improvement was observed in hydration score, total wrinkle count, fine lines count, and average wrinkle severity compared to the placebo (n = 8) [27]. In both ZO-1 and ZO-2 groups, there were multiple ingredients in addition to zeaxanthin which may or may not have confounded the results of this study.

Capsule summary: Evidence supports enhanced photoprotection, beneficial changes in skin pigmentation, hydration, and wrinkle appearance with zeaxanthin supplementation. Lutein supplementation has been shown to improve photoprotection and pigmentation. Lastly, supplementation with lycopene improves photoprotection, wrinkle appearance, hydration, texture, and elasticity.

Paprika

Yatsuhashi et. al. conducted a four-week, randomized, single-blind, parallel-group controlled trial to study the effects of oral supplementation with 9 mg of paprika xanthophyll capsules daily. Results demonstrated statistically significant improvements in moisture in the treatment group (n = 12) compared to the placebo (n = 12). There was no difference between the treatment group and placebo regarding elasticity and wrinkle appearance [28].

Capsule summary: Evidence supports dietary supplementation with paprika improves skin hydration.

Small molecules

Glutathione

Glutathione is a small molecule composed of cysteine, glutamine, and glycine. Reduced glutathione (GSH) is more prevalent than the disulfide form (GSSG) within healthy cells, and measuring this ratio can help indicate the presence of oxidative stress. As an oral supplement, glutathione is commonly used to lighten skin color [29].

A 12-week, randomized, double-blind study compared placebo to L-cystine, GSH, and a combination of L-Cys and GSH. Hard shell capsules were ingested twice daily by placebo (n = 32), 500 mg L-Cys (n = 30), and a combination of both 500 mg L-cystine and 250 mg GSH (n = 31) groups. Tablets were ingested once daily for the 250 mg GSH (n = 31) group. Regarding both tablets and capsules being used in this study, the authors stated, “The galenic form was different because a commercial product was used as a comparator. However, the investigated treatments were presented to the subjects and investigators in a blinded format to ensure the respect of the double-blind nature of the study” [29]. This study found L-cystine and L-glutathione combination significantly lightened dark spots compared to the other three groups. Authors concluded that L-cystine may have a synergetic whitening effect with the reduced form of glutathione [29].

Weschawalit et al. conducted a double-blind, placebo-controlled, parallel, three-arm study in which middle-aged, female subjects took 250 mg of GSSG (n = 18), GSH (n = 20), or placebo (n = 19) once nightly for 12 weeks. This study demonstrated that the TEWL of the GSH group was significantly lower than the GSSG group and placebo; wrinkle formation was significantly lower in the GSH group compared to the placebo; the melanin index was significantly lower in the GSH group compared to the placebo; and while not statistically significant, elasticity was notably higher in the GSSG and GSH group compared to placebo [30]. No serious adverse events occurred, but some adverse reactions included pruritus, macular erythema, and fatigue in the treatment group.

Capsule summary: Evidence supports improvements in skin hydration, pigmentation, and wrinkle appearance with supplementation of GSH.

Aloe sterols

Aloe vera contains sterols that have been found to activate peroxisome proliferator-activated receptors (PPARs) which regulate gene expression of proteins involved in anti-inflammatory pathways [31]. PPAR signaling has been associated with the downregulation of MMPs in mice induced with UV radiation. These mechanisms may play a role in the anti-aging effects of the sterols found in aloe.

After a 12-week, double-blind, randomized controlled study, supplementation with drinkable yogurt containing 0.5 g aloe gel powder (40 µg of aloe sterols per 100 g) was found to significantly reduce TEWL and increase collagen and elasticity (n = 60) compared to the placebo group (n = 58). This suggests that aloe sterols improve skin barrier function and skin hydration [31].

Cho et al. demonstrated how dissolving either 1% aloe vera powder (n = 15) in 120 mL of distilled water (equivalent to 1,200 mg of aloe vera gel per day) or 3% (n = 15) in 120 mL (3,600 mg gel per day) for 90 days can significantly decrease facial wrinkles and skin surface roughness. The 1% group demonstrated increased cutaneous elasticity compared to the 3% treatment group. This study showed how dietary aloe supplementation improved elasticity and clinical improvement of facial wrinkles [32].

In a double-blinded, randomized, placebo-controlled study, five tablets with a total of 40 mg of aloe sterols taken daily for 12 weeks (n = 24) significantly increased skin elasticity compared to tablets containing dextrin in the placebo group (n = 24) [33].

Capsule summary: Evidence supports that aloe sterol supplementation improves skin texture, wrinkle appearance, elasticity, and hydration.

Melon concentrate

Superoxide dismutase (SOD) is a widely studied, plant-derived enzyme with antioxidant capabilities found in melons. SODB is a dried melon juice concentrate from *Cucumis melo L*., which is particularly rich in SOD. For oral administration, the concentrate is coated in palm oil to preserve its activity and contains other antioxidants, including glutathione, carotenoids, coenzyme Q10, vitamin C, and vitamin E. One capsule containing 20 mg of SODB (280 U of SOD) was taken daily for 32 days. There was a significant difference in an increased MED in the supplement group (n = 22) compared to the placebo group (n = 22) [34].

Capsule summary: Melon concentrate appears to have photoprotective effects, but the evidence is limited by a lack of studies.

Summary

Plant-derived dietary supplements reviewed in this article and their effects on skin health parameters, including photoprotection, pigmentation, texture, wrinkle appearance, hydration, elasticity, and microcirculation, are summarized in Table 1.

**Table 1:** Plant-derived antioxidants and their effects on skin parameters.

	Improved photoprotection	Improved pigmentation	Improved texture	Improved wrinkle appearance	Improved hydration	Improved elasticity	Improved microcirculation	References
Pine bark extract	X	X	X		X	X		[4-7]
Cocoa	X		X	X			X	[8-11]
Turmeric								[12-14]
Polypodium leucotomos	X	X			X	X		[15-18]
Ferulic acid					X		X	[19,20]
Astaxanthin	X	X	X	X	X	X		[22,23]
Lutein	X	X						[24,25]
Zeaxanthin	X	X	X	X	X			[24,27]
Lycopene	X	X	X	X	X	X		[25,26]
Paprika					X			[28]
Glutathione		X		X	X			[29,30]
Aloe sterols			X	X	X	X		[31,33]
Melon concentrate	X							[34]

## Conclusions

Consumers should be aware that the FDA does not regulate oral supplementation products and companies are not required to provide clinical data on each product’s efficacy, safety, or subsequent effects on the body. Although evidence-based studies are needed to provide patients with accurate information and useful recommendations for oral supplements, increased advocacy for FDA regulation of dietary supplements is needed. There are various plant-derived, antioxidant supplements with supporting evidence for improved skin health and/or signs of aging. However, many supplements are lacking multiple studies evaluating their effects on the skin. This review evaluated the supplements and their effects on skin pigmentation, texture, wrinkle appearance, hydration, elasticity, and microcirculation. Further studies with larger sample sizes are needed to assess the effects of popular supplements covered in this review to better aid patients in choosing the best oral supplement for their skin needs.
